# Long COVID in Uganda: Electrographic findings among patients at risk

**DOI:** 10.1002/puh2.78

**Published:** 2023-04-04

**Authors:** Andrew Weil Semulimi, Charles Batte, Daniel Iraguha, Pamela Apio Okwir, Hope Atuhaire, Chelsea Lipoto, Tonny Muwonge, Norah Namirembe, Grace Biyinzika Lubega, Provia Ainembabazi, John Mukisa, Felix Bongomin, Isaac Ssinabulya, Emmy Okello

**Affiliations:** ^1^ Lung Institute, Department of Medicine, School of Medicine College of Health Sciences, Makerere University Kampala Uganda; ^2^ Division of Adult Cardiology Uganda Heart Institute Kampala Uganda; ^3^ Clinical Epidemiology Unit, School of Medicine, College of Health Sciences Makerere University Kampala Uganda; ^4^ Department of Medicine, School of Medicine, College of Health Sciences Makerere University Kampala Uganda; ^5^ Department of Epidemiology and Biostatistics, School of Public Health College of Health Sciences, Makerere University Kampala Uganda; ^6^ Department of Research Infectious Diseases Institute Kampala Uganda; ^7^ Department of Molecular Biology and Immunology, School of Biomedical Sciences, College of Health Sciences Makerere University Kampala Uganda; ^8^ Department of Medical Microbiology and Immunology Faculty of Medicine, Gulu University Gulu Uganda

**Keywords:** Africa, cardiac arrhythmias, cardiac conduction system disease, Long COVID, myocardial ischemia, pandemic, post‐acute COVID‐19 syndrome, Uganda

## Abstract

**Background:**

COVID‐19 has a significant cardiovascular involvement. An electrocardiographic (ECG) abnormalities among people at a risk of Long COVID in Uganda was investigated.

**Methods:**

A cross‐sectional study was conducted from February to June 2022 at the post COVID‐19 clinic in Mulago National Specialized Hospital, Kampala. A standard resting ECG was performed on individuals at least 2 months following acute COVID‐19, with a negative SARS‐CoV‐2 reverse‐transcription polymerase chain reaction. Socio‐demographic and clinical characteristics as well as vital signs were recorded for all study participants.

**Results:**

Of the 244 study participants, 117 (47.9%) were female. The median age of all the participants was 33.0 (interquartile range: 26.0–43.5) years. Twenty‐five (10.2%) participants had a history of smoking, whereas 117 (48%) had a history of alcohol intake. In total, 46 (18.9%) had abnormal ECG findings (95% Confidence Interval [CI]: 14.39–24.29), and nonspecific T‐wave inversion (*n* = 16, 34%) was the most frequent ECG abnormality. The proportion of participants with ECG abnormalities was 48% lower among females (adjusted prevalence ratio [aPR]: 0.52, 95% CI: 0.28–0.96, *p* value <0.05) and twofold greater for those with a history of smoking (aPR: 2.03, 95% CI: 1.096–3.776, *p* value <0.05).

**Conclusion:**

One in five Ugandans who were checked at the clinic at a risk of Long COVID showed ECG abnormalities. ECG screening is suggested to be integrated into the follow‐up care of those at a risk of Long COVID.

## Graphical abstract

Key Findings:

Individuals at risk of Long COVID‐19 might present with symptoms of cardiovascular diseases (CVDs).

Almost 20% of individuals at a risk of Long COVID‐19 had an ECG abnormality and individuals with a history of smoking had a twofold greater risk of developing an ECG abnormality.

Screening for CVDs among individuals at a risk of Long COVID‐19 should be incorporated in their clinical care.

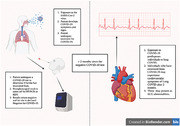

## INTRODUCTION

Vaccination against COVID‐19 has significantly reduced mortality and morbidity [1]. COVID‐19 is a multisystemic disease [2] with cardiovascular system complications. This may persist even after recovery resulting into symptoms of Long COVID [3]. A high proportion of people at risk of Long COVID developed cardiovascular diseases (CVDs) [4], especially during the convalescent phase [5]. As countries rolled out vaccination programs against COVID‐19, which caused a reduction in the COVID‐19 cases globally, identification of long‐term consequences of COVID‐19‐related CVDs is essential in guiding plans to address the developing burden. CVDs risk assessment and screening are a key strategy to establish the CVDs burden among people at risk of Long COVID. Electrocardiograms (ECGs) are inexpensive and widely available in many developing countries like Uganda and could be used to routinely monitor and assess long‐term cardiovascular complications of COVID‐19. However, data to guide the formulation of guidelines for routine use of ECGs to screen for CVDs among people at a risk of Long COVID is limited. This study looked at ECG abnormalities among individuals at risk of Long COVID and understand their predictors.

## METHODS

### Study design, site, and population

Between February and June 2022, a cross‐sectional study was conducted among individuals at risk of Long COVID who were recruited at the Mulago National Specialized Hospital (MNSH) which managed over 1000 cases of COVID‐19. MNSH post COVID‐19 clinic, the recruitment site, was one of the three specialized post COVID‐19 clinics in Kampala, which received an estimate of 30 patients per day [6] until its services were incorporated into the normal specialized clinics of the hospital. A simple random sampling was used to recruit participants who were 18 years and above and presented at the clinic more than 2 months after recovery from the COVID‐19 with a negative reverse‐transcription polymerase chain reaction. Participants who reported with preexisting comorbidities, such as diabetes mellitus, heart failure, hypertension, and those taking drugs which could alter ECG findings were excluded.

### Study variables and data collection

Research assistants administered a questionnaire which explored socio‐demographics characteristics such as age, and clinical history such as duration of COVID‐19 illness, as well as CVDs risk factors such as history of smoking. Height and weight were measured using a calibrated length scale and manual SECA 750 adult weighing scale to calculate Body Mass Index (BMI). Data on ECG was collected using a resting 12‐lead ECG and a Schiller at 102 resting ECG machine (Schiller, Switzerland). Each ECG result was interpreted independently by two cardiologists. The Minnesota coding system that is a manual to guide the classification of electrocardiographic morphology [7] was modified though this manual does not provide guidance on manual reporting of ECG results.

### Data analysis

Data was analyzed using STATA 16.0 (StataCorp LLC, College Station, TX, USA). Participant characteristics were presented as frequencies and percentages for categorical data and medians and interquartile ranges for nonparametric variables. BMI was categorized as underweight (≤18.4 kg/m^2^), normal (18.5–24.9 kg/m^2^), overweight (25.0–29.9 kg/m^2^), and obese (≥30 kg/m^2^) [8]. Blood pressure was classified as normal if systolic blood pressure (SBP) was 120–139 mm Hg, and/or diastolic blood pressure (DBP) was 60–89 mm Hg and considered abnormal based if SBP was ≥140 mm Hg and/or DBP of ≥90 mm Hg for sitting blood pressure measurement [9]. A decrease in SBP of at least 20 mm Hg or a decrease in DBP of at least 10 mm Hg was considered abnormal standing blood pressure.

The proportions of people at a risk of Long COVID with different ECG presentations were expressed as percentages and visualized as pie chart. Associations between ECG abnormalities and independent variables were assessed using Pearson's Chi‐squared or Fisher's exact tests for categorical data. Numerical variables were compared using student *t*‐test for parametric and Mann–Whitney *U* test for nonparametric variables. Prevalence ratios were chosen because the prevalence of the outcome was more than 10% and were calculated by fitting modified Poisson regression models with robust standard errors. The adjusted ratios were determined by fitting models with different exposure variables in the same model basing on variables that had previously been shown to affect ECG presentations in COVID‐19 for instance sex of the participant, BMI, history of smoking and alcohol use, and duration of the hospitalization (a proxy for disease severity). A *p* value <0.05 was considered statistically significant.

### Ethical considerations

The study was approved by the Mulago Hospital Research and Ethics Committee of Makerere University (MHREC 2021‐52) and by the Uganda National Council for Science and Technology (HS1974ES). Participants diagnosed with any ECG abnormality such as atrial fibrillation were referred to a cardiologist for further medical attention. ECGs were conducted in a private room in the presence of a chaperone for female participants. A research assistant conducted ECG procedures and written informed consent was obtained before the ECG was done.

## RESULTS

### Participant characteristics

**FIGURE 1:**
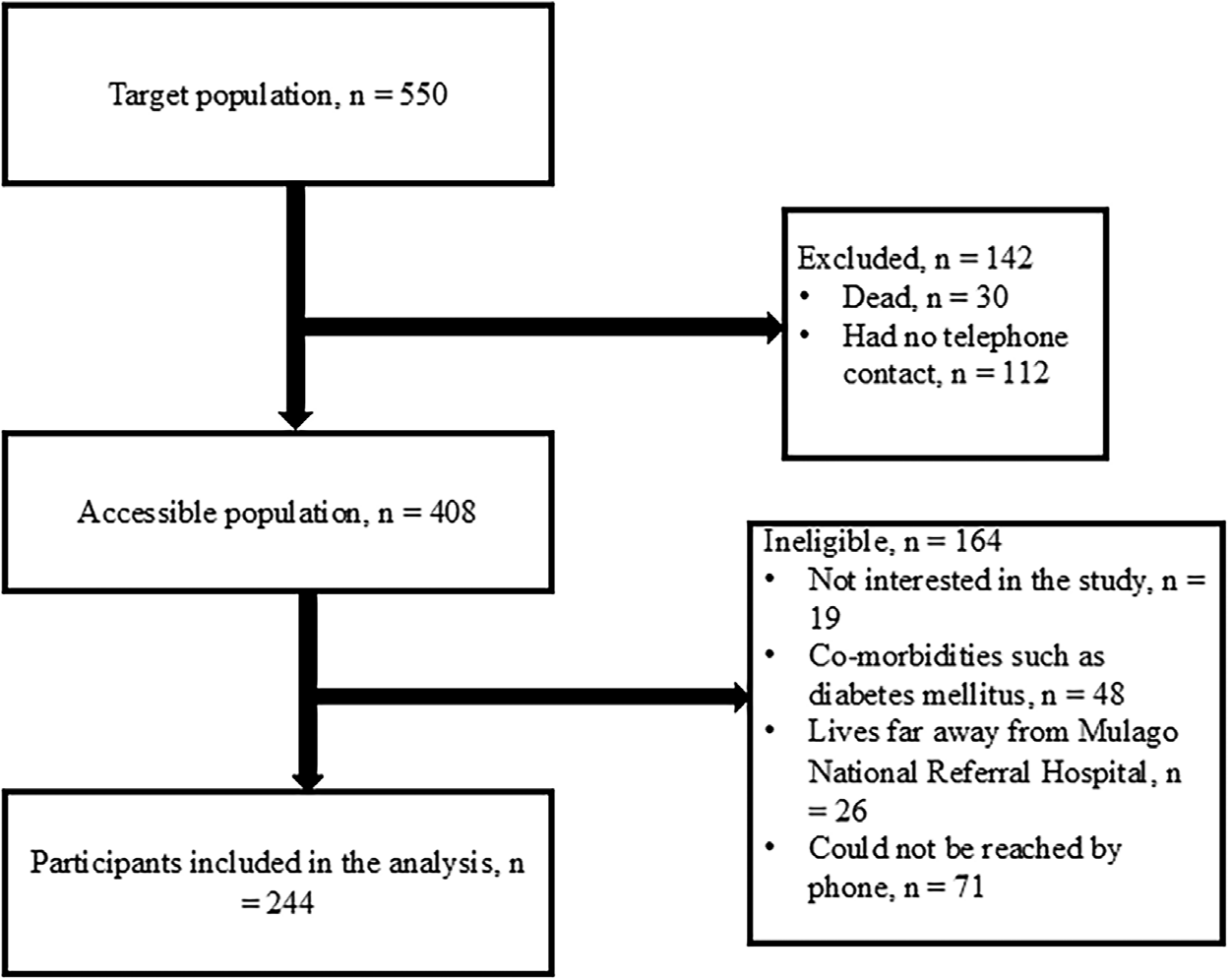
The flow of participants at a risk of Long COVID who took part in the electrocardiographic (ECG) assessment in Kampala, Uganda, February–June 2022.

A total of 244 eligible participants were enrolled in the study. Figure 1 shows the median age at 33 (IQR: 26–43.5) years, with 117 (47.9%) female participants as shown in Table 1. Fatigue (65, 67.6%) and chest pain (130, 53.3%) were the most common symptoms. Overall, 103 (42.2%) participants had moderate form of COVID‐19 and 21 (45.6%) of whom had ECG abnormalities.

**TABLE 1:** The socio‐demographics and clinical history of people at a risk of Long COVID in Kampala, Uganda from February–June 2022.

Variable	Normal ECG (*n* = 198), *n* (%)	Abnormal ECG (*n* = 46), *n* (%)	Total, *n* (%)
**Age** **^a^**			
≤33 years	103 (52.0)	21 (45.7)	124 (50.8)
≥34 years	95 (48.0)	25 (54.3)	120 (49.2)
**Sex**			
Male	94 (47.5)	33 (71.7)	127 (52.1)
Female	104 (52.5)	13 (28.3)	117 (47.9)
**Marital status**			
Not married	101 (51.0)	19 (41.3)	120 (49.2)
Married	97 (49.0)	27 (58.7)	124 (50.8)
**Income status in USD** ^b^			
<13.5	71 (35.9)	21 (45.6)	92 (37.7)
>14–27	48 (24.2)	8 (17.4)	56 (23.0)
>28	79 (39.9)	17 (37.0)	96 (39.3)
**Clinical history**			
**Include reasons for coming to the post COVID‐19 clinic** **^c^**			
Fatigue	138 (69.7)	27 (58.7)	165 (67.6)
Chest pain	109 (55.1)	21 (45.6)	130 (53.3)
Difficulty in breathing	84 (42.4)	21 (45.6)	105 (43.0)
Palpitations	58 (29.3)	14 (30.4)	72 (29.5)
Joint pains	88 (44.4)	20 (43.5)	108 (44.3)
None	25 (13.8)	7 (15.2)	32 (14.1)
**Duration of symptoms from diagnosis to discharge from care (Negative RT—PCR)**			
≤14 days^d^	194 (98.0)	45 (97.8)	239 (98.0)
>14 days	4 (2.0)	1 (2.2)	5 (2.0)
**Form of COVID‐19**			
Mild	86 (43.4)	20 (43.5)	106 (43.4)
Moderate	82 (41.4)	21 (45.6)	103 (42.2)
Severe	30 (15.2)	5 (10.9)	35 (14.3)
**Type of management of COVID‐19**			
Home‐based care	108 (54.5)	22 (47.8)	130 (53.2)
Hospital‐based management	90 (45.5)	24 (52.2)	114 (46.7)

^a^ Age categories are based on the median value.

^b^ United States Dollar (USD) = 3700 Uganda Shillings.

^c^ Participants had multiple symptoms.

^d^ Based on the published clinical duration of COVID‐19.

### Cardiovascular diseases risk factors

In total, 25 (10.2%) participants had a history of smoking. Among this, 10 (21.7%) had ECG abnormalities as shown in Table 2. Overall, 177 (72.5%) participants had a history of alcohol intake of whom 24 (52.2%) had ECG abnormalities.

**TABLE 2:** The distribution of cardiovascular risk factors among individuals at a risk of Long COVID in Kampala, Uganda from February–June 2022.

Variable	Normal ECG (*n* = 198), *n* (%)	Abnormal ECG (*n* = 46), *n* (%)	Total, *n* (%)
**History of smoking**	15 (7.6)	10 (21.7)	25 (10.2)
**History of alcohol intake**	93 (47.0)	24 (52.2)	117 (47.9)
**Body Mass Index (kg/m^2^)**			
Underweight (≤18.4)	2 (1.0)	2 (4.3)	4 (1.6)
Normal (18.5–24.9)	83 (41.9)	17 (37.0)	100 (41.0)
Overweight (25.0–29.9)	68 (34.3)	18 (39.1)	86 (35.2)
Obese (≥30)	45 (22.7)	9 (19.6)	54 (22.1)
**Abnormal sitting Blood pressure (mm Hg)** ^a^	9 (4.5)	7 (15.2)	16 (6.6)
**Abnormal standing blood pressure (mm Hg)** ^b^	22 (11.1)	14 (30.4)	36 (14.8)
**Activity level post discharge**			
Sedentary/mild exercise	142 (71.7)	36 (78.3)	178 (73.0)
Moderate/strenuous exercise	56 (28.3)	10 (21.7)	66 (27.1)
**Sad, blue, depressed post COVID‐19**	75 (37.9)	17 (36.9)	92 (37.7)

^a^ Systolic blood pressure (SBP) of ≥140 mm Hg and/or diastolic blood pressure (DBP) of ≥90 mm Hg.

^b^ A decrease in SBP of at least 20 mm Hg or a decrease in DBP of at least 10 mm Hg.

### Participants with ECG abnormalities

**FIGURE 2:**
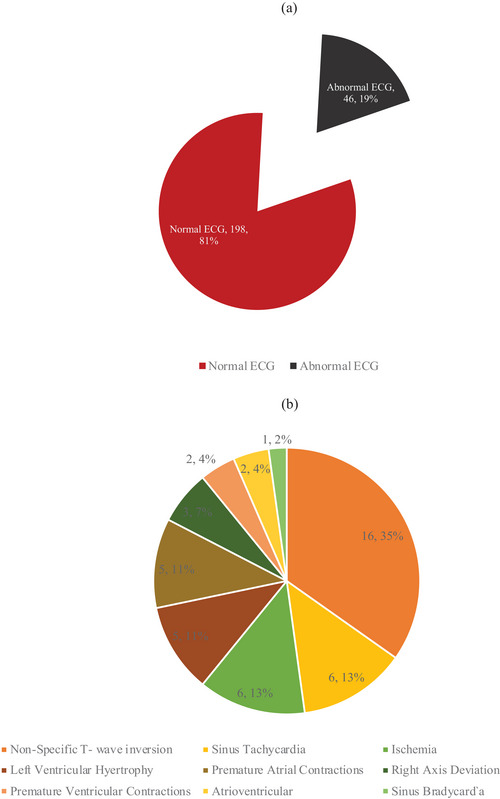
The patterns of electrocardiographic (ECG) findings among people at a risk of Long COVID in Kampala, Uganda from February–June 2022. (a) The proportion of people at a risk of Long COVID with ECG abnormalities in Kampala, Uganda from February–June 2022. (b) The distribution of ECG abnormalities among people at a risk of Long COVID, *n* = 46, in Kampala, Uganda from February–June 2022.

Overall, 46 (19%) participants had ECG abnormalities as presented by Figure 2. The most common ECG abnormality was nonspecific T‐wave inversion (16, 34.8%), frequently in the aVL lead (8/16, 50%) as shown in Table 3.

**TABLE 3:** The distribution of electrocardiographic (ECG) abnormalities among participants at a risk of Long COVID in Kampala, Uganda from February to June 2022.

Variable	Frequency (*n* = 46)	Percentage
**Type of abnormality**		
Nonspecific T‐wave inversion	16	34.8
Sinus tachycardia	6	13
Ischemia	6	13
Left ventricular hypertrophy	5	10.9
Premature atrial contractions	5	10.9
Right axis deviation	3	6.5
Premature ventricular contractions	2	4.3
Atrioventricular block	2	4.3
Sinus bradycardia	1	2.2
**Nonspecific T‐wave inversion categories**		
T‐wave inversion in lead AVL	8	50
T‐wave inversion in lead III	7	41.8
T‐wave inversion in lead III and AVF	1	6.4

### Predictors of ECG abnormalities

At multivariate analysis, the proportion of participants with ECG abnormalities was 48% lower among females (adjusted prevalence ratio (aPR): 0.52, 95% CI: 0.28–0.96, *p* value <0.05) and twofold greater for participants with a history of smoking (aPR: 2.03, 95% CI: 1.096–3.776, *p* value <0.05) as presented in Table 4.

**TABLE 4:** The predictors of electrocardiographic (ECG) abnormalities among participants at a risk of Long COVID in Kampala, Uganda from February to June 2022.

Variable	Prevalence ratio (95% CI)	*p* Value	Adjusted prevalence ratio (95% CI)	*p* Value
**Age**				
≤33 years	1			
≥34 years	1.23 (0.73–2.08)	0.44		
**Sex**				
Female	0.43 (0.24–0.77)	0.005	0.52 (0.28–0.96)	0.037
**Marital status**				
Married	1.37 (0.81–2.34)	0.24		
**Income status in USD** ^a^				
<13.5	1			
14–27	0.62 (0.3–1.32)	0.22		
>28	0.77 (0.44–1.37)	0.39		
**Duration of symptoms from diagnosis to discharge from care**				
≤14 days	1			
>14 days	1.08 (0.12–9.88)	0.95		
**Form of COVID**—**19**				
Mild‐	1			
Moderate	1.08 (0.62–1.87)	0.78		
Severe	0.757 (0.31–1.87)	0.55		
**Type of management of COVID‐19**				
Home‐based care	1			
Hospital‐based management	1.31 (0.69–2.45)	0.41		
**History of smoking**	2.43 (1.38–4.29)	0.002	2.03 (1.1–3.78)	0.024
**History of alcohol intake**	1.18 (0.70–2)	0.53		
**BMI (kg/m^2^)**				
Underweight (≤18.4)	2.94 (1.0–8.61)	0.05		
Normal (18.5–24.9)	1			
Overweight (25.0–29.9)	1.23 (0.68–2.24)	0.5		
Obese (≥30)	0.98 (0.47–2.05)	0.96		
**Abnormal sitting Blood pressure (mm Hg)**	2.56 (1.37–4.78)	0.003	1.69 (0.85–3.34)	0.131
**Abnormal standing blood pressure (mm Hg)**	2.52 (1.50–4.25)	< 0.001	1.78 (0.97–3.25)	0.06
**Activity level post discharge**				
Sedentary/mild exercise	1			
Moderate/strenuous exercise	0.704 (0.33–1.51)	0.37		
				
**Sad, blue, depressed post COVID‐19**	0.97 (0.56–1.66)	0.908		

^a^ USD: United States Dollars.

## DISCUSSION

This study aimed at investigating the presence of ECG abnormalities among participants following an acute episode of COVID‐19. The study found almost one in five of the study participants having at least one ECG abnormality. ECG abnormalities were almost 50% lower among females compared to male participants. A history of smoking showed a twofold higher risk of having an ECG abnormality compared to participants who are nonsmokers. These findings are in agreement with prior studies [5, 10, 11] which suggest the presence of ECG abnormalities among individuals at risk of Long COVID. This is probably the first study to determine the prevalence of ECG abnormalities among survivors of COVID‐19 without preexisting comorbidities prior to the acute episode of COVID‐19. The difference in the proportions could be explained by the consideration of participants with preexisting comorbidities such as hypertension, the age of the participants and the duration after discharge from hospital.

Similar to a Polish Long COVID cardiovascular study [3], most of the participants in this Uganda study presented with complaints of fatigue, chest pain, difficulty in breathing, and palpitation which would warrant further CVD assessment which include ECG to rule out myocardial infarctions, life threatening arrhythmias, or any conduction abnormality. Coincidentally, 13% of the participants were found to have ischemic ECG features, whereas 34.8% of them had nonspecific T‐wave invasion. Although T‐wave inversion has been considered a minor nonspecific clinical finding, it could highlight underlying cardiac remodeling or acute coronary syndrome and predict acute coronary syndrome and sudden cardiac death [12, 13]. The presence of right axis deviation may demonstrate underlying pulmonary involvement especially venous thromboembolism and pulmonary embolism [14].

This study in Uganda suggests that ECG abnormalities were lower among females, which differs from findings from a cohort study in China [4]. More females than males presented with symptoms of Long COVID which included chest pain, difficulty in breathing, and palpitations that are hallmarks of CVDs [3]. This could have prompted individuals to seek care at post COVID‐19 clinic leading to the initiation of treatment arresting the progression of the sequalae. ECG abnormalities were twofold greater among smokers. Individuals with history of smoking had elevated risk of hospitalization, increased disease severity, and mortality from COVID‐19 [15] due to its destructive effects on the lung, cardiomyocytes, and vascular endothelium [16]. The dual presence of the long‐term effects of smoking and COVID‐19 may have exacerbated the risk of developing ECG abnormalities among the participants in this study. Other CVD risk factors such as obesity, history of alcohol intake, age, and sedentary lifestyle had not statistical significance in this study. This may be due to the age of the study population that was younger and active with normal BMI which could lower the risk of CVDs.

One of the limitations of this study is the lack of baseline ECG measures of the participants before and during the acute phase of COVID‐19, thus no comparison could be done which would have strengthened the study findings. The study is unable to rule out biochemical factors such as electrolyte abnormalities that could influence the results of the ECGs. The study also included participants who received treatment from tertiary hospitals and findings may not be generalizable to other clinical settings. However, the study provides a crucial preliminary data that could guide the development of management frameworks of Long COVID and provide a basis for future research. The study only enrolled participants without preexisting comorbidities and those within the convalescent stage which was more than 2 months after discharge from the hospital with a negative COVID‐19 test which ruled out potential confounders.

## CONCLUSION

This study in Uganda presents substantial evidence on the possible effects of an acute episode of COVID‐19 on the cardiovascular system. The ECG abnormalities found among participants who had recovered from COVID‐19 contributes to our understanding of the potential impact of Long COVID. Policy makers and governments should provide more funding for research on Long COVID, whereas clinicians should develop frameworks and guidelines for continued monitoring of individuals at a risk of Long COVID. So, we look forward to conducting further studies among individuals at a risk of Long COVID across time for the scientific and health community to better prepare for this rising and yet silent epidemic.

## AUTHOR CONTRIBUTIONS

Andrew Weil Semulimi, Charles Batte, Provia Ainembabazi, Grace Biyinzika Lubega, John Mukisa, Felix Bongomin, Isaac Ssinabulya, and Emmy Okello took part in the design and conduct of the study. Chelsea Lipoto, Pamela Apio Okwir, Tonny Muwonge, Hope Atuhaire, Daniel Iraguha, and Norah Namirembe carried out the data collection and entry process. John Mukisa, Andrew Weil Semulimi, Felix Bongomin, Provia Ainembabazi, Pamela Apio Okwir, Daniel Iraguha, and Charles Batte analyzed and interpreted the study data. Andrew Weil Semulimi and Pamela Apio Okwir wrote the first draft of the manuscript. All authors read, reviewed, and approved the final manuscript.

## CONFLICT OF INTEREST STATEMENT

The authors declare they have no conflict of interests.

## FUNDING INFORMATION

The study was supported by National Institute of Health Research through the Royal Society of Medicine and Hygiene (RSTMH) 2021 Early Career grants. Additional funding for the study is from the Makerere University Non‐Communicable Diseases (MAKNCD) Research Training Program with support from the Fogarty International Center of the National Institutes of Health under Award Number D43TW011401. The content is solely the responsibility of the authors and does not necessarily represent the officials views of the funders. The funders had no role in the study design, data collection, analysis and decision to publish or in the preparation of the manuscript.

## Supporting information

Supporting Information

Supporting Information

## Data Availability

The datasets used and/or analyzed during the current study have been attached as a Supporting Information section 1 and 2.
