# Supplementary material for: Long COVID in Uganda: Electrographic findings among patients at risk
Source: Public Health Chall. 2023 Apr 4;2(2):e78. doi: 10.1002/puh2.78 (PMC10586224; doi:10.1002/puh2.78)
Supplement: Supplementary file 1 — Supporting Information [file PUH2-2-e78-s001.docx]

Supplementary file 1: Code labels for study participant in the Supplementary file 2.

| **Variable** | **Category** | **Code** |
| --- | --- | --- |
| Age | ≤33 | 1 |
|  | ≥34 | 2 |
| Sex |  |  |
|  | Male | 1 |
|  | Female | 2 |
| Marital status |  |  |
|  | Not married | 1 |
|  | Married | 2 |
| Income status in UGX |  |  |
|  | <50,000 | 1 |
|  | >50,000-100000 | 2 |
|  | >100000 | 3 |
| Fatigue |  |  |
|  | No | 0 |
|  | Yes | 1 |
| Chest pain |  |  |
|  | No | 0 |
|  | Yes | 1 |
| Difficulty in breathing |  |  |
|  | No | 0 |
|  | Yes | 1 |
| Palpitations |  |  |
|  | No | 0 |
|  | Yes | 1 |
| Joint pains |  |  |
|  | No | 0 |
|  | Yes | 1 |
| Fever |  |  |
|  | No | 0 |
|  | Yes | 1 |
| Abdominal pain |  |  |
|  | No | 0 |
|  | Yes | 1 |
| Duration of symptoms from diagnosis to discharge from care |  |  |
|  | ≤ 14 days | 0 |
|  | >14 days | 1 |
| Form of Covid-19 |  |  |
|  | Mild- | 1 |
|  | Moderate | 2 |
|  | Severe | 3 |
| Type of management of Covid-19 |  |  |
|  | Home based care | 1 |
|  | Hospital based management | 2 |
| History of smoking |  |  |
|  | Yes | 1 |
|  | No | 2 |
| History of alcohol intake |  |  |
|  | Yes | 1 |
|  | No | 2 |
| Activity level post discharge |  |  |
|  | Sedentary/mild exercise | 1 |
|  | Moderate/strenuous exercise | 2 |
| Sad, blue, depressed post COVID-19 |  |  |
|  | Yes | 1 |
|  | No | 2 |
| BMI |  |  |
|  | Normal | 1 |
|  | Underweight | 2 |
|  | Overweight | 3 |
|  | Obese | 4 |
| Sitting Blood pressure |  |  |
|  | Abnormal | 1 |
|  | Normal | 2 |
| Standing Blood pressure |  |  |
|  | Abnormal | 1 |
|  | Normal | 2 |
| ECG results |  |  |
|  | Normal | 0 |
|  | Abnormal | 1 |
| Abnormal ECG |  |  |
|  | Non-specific TWI | 3 |
|  | Sinus Tachycardia | 6 |
|  | Ischemia | 7 |
|  | LVH | 2 |
|  | PACs | 4 |
|  | Right Axis Deviation | 8 |
|  | PVCs | 5 |
|  | AV block | 1 |
|  | Sinus Bradycardia | 9 |

AV- Atrioventricular, BMI- Body Mass Index, BP- Blood Pressure, BM- Beats per minutes, ECG- Electrocardiogram, LVH- Left Ventricular Hypertrophy, PVC- Premature Ventricular Contractions, PAC- Premature Atrial Contraction.
